# Application of Composite Gels in Food Processing and Engineering

**DOI:** 10.3390/gels12080737

**Published:** 2026-08-18

**Authors:** Lei Zhou, Xiaoyan Hu

**Affiliations:** 1 College of Food Science and Technology, Hunan Agricultural University, Changsha 410114, China; 2 Biopolymers and Colloids Laboratory, Department of Food Science, University of Massachusetts, Amherst, MA 01003, USA; xh897722@gmail.com

## 1. Introduction

Foods are complex structures typically composed of proteins, polysaccharides, and fats. These components, especially proteins and polysaccharides, form a three-dimensional gel network structure under various processing conditions such as heating, pH, pressure, metal ions, or enzymes, thus imparting the desired sensory properties to different products [1]. Whether traditional foods like emulsified sausages or novel foods like plant-based protein meat products, they are all composite gel systems. Therefore, with the advancement of food processing technology and consumer demand for novel products, more and more researchers are focusing on composite gel systems in food [2,3]. However, due to the multi-component nature of composite gel systems, their properties are influenced by various factors. This presents many challenges to the adaptability of composite gel systems in different food processing applications [4,5].

With increasing consumer demand for non-animal protein, many non-traditional gel matrices (such as plant proteins or polysaccharides) are being incorporated more frequently into composite gel systems. Therefore, understanding the impact of these components on the properties of traditional composite gels is crucial for designing novel gelled foods. The formation conditions of composite gels are a significant factor influencing their quality. The formation conditions of composite gels change with changes in food processing and application conditions, resulting in different gel properties. Furthermore, the production of composite gel products often involves various food processing steps, especially for non-traditional foods, which can significantly affect the properties of the composite gel and thus affect the product quality. Therefore, monitoring changes in the properties of composite gels under different conditions is an important way to increase their processing adaptability and product quality.

The formation of composite gel systems is a complex process. For composite gel systems, the changes in each component affect the textural and sensory characteristics of the final product. Therefore, it is important to consider the question of how to precisely control the molecular properties of composite gels to produce satisfactory products. Although traditional gel evaluation methods, such as gel properties and sensory properties, are often used to evaluate the properties of composite gels, they often damage the gel. Therefore, establishing advanced gel evaluation methods to adapt to modern industrial production is crucial for the production of high-quality composite gel products.

Thus, considering the various problems currently existing in composite gel systems, the journal *Gels* launched a Special Issue, “Application of Composite Gels in Food Processing and Engineering”, to collect current progress on the application of composite gels in food systems. This Special Issue includes seven cutting-edge studies on composite gels in the food field (six research papers and one review). These studies cover aspects such as gel formation methods, the application of novel gel matrices, novel processing methods, novel products, and novel gel evaluation methods. In the following sections, we summarize in detail the main objectives, content, innovative points, and how these different publications broaden the application of composite gel systems in the food industry.

## 2. Overview of the Published Papers in This Special Issue

The article “Effect of Curdlan on the Structural Stability and Thermal Processing Properties of Mycelium-Based Gels Used in 4D-Printed Meat Analogs” by Hu et al. studied the effects of polysaccharide addition on the quality of 4D-printed meat analogs. To overcome the structural instability and poor thermal processing properties of Pleurotus eryngii mycelium–soy protein isolate–cassava starch composite gels as meat protein substitutes, Hu et al. investigated the impact of different concentrations of curdlan (0–5%, w/w) on the gel structure, rheological behavior, 3D printing performance, water distribution, and thermal processing properties of this composite gel. Their results demonstrated that curdlan significantly enhanced the mechanical stability of the composite gel system while maintaining perfect printing performance, with optimized formulations achieving high structural retention (>87%). During thermal processing, curdlan acted as an internal supporting network, reducing structural collapse, water loss, and shrinkage. This article extends the use of polysaccharides from traditional applications, such as texture improvement, stabilization, and encapsulation, toward advanced food manufacturing technologies, particularly 4D food printing and alternative protein production.

The paper “Regulation of Microstructure and Properties of Konjac Glucomannan Gels via Ethanol Under Low-Alkali Conditions” by Xu et al. investigated the effect of the gel-forming environment on the gel-forming ability of commonly used polysaccharides. To overcome the limitations of traditional alkali-induced konjac glucomannan gels, which usually require strong alkaline environments (pH 11–13) that may cause undesirable alkaline flavors, excessive water loss, environmental concerns, and reduced suitability for food applications, Xu et al. evaluated the influence of ethanol addition (0–20%) on the structural and functional properties of konjac glucomannan gels. They found that the properties of the konjac glucomannan gel gradually improved with increasing ethanol concentration. However, excessive ethanol addition (20%, *v*/*v*) induced local molecular aggregation and disrupted the uniformity of the gel network. Among the tested conditions, 15% ethanol provides an optimal balance between molecular interactions and network integrity, producing konjac glucomannan gels with compact microstructures, enhanced deformation resistance, and improved thermal stability. This study expands the application potential of composite gel systems in the food industry by enhancing the practicality and functionality of konjac glucomannan-based gels.

The third article, “The Effect of Fructooligosaccharide and Inulin Addition on the Functional, Mechanical, and Structural Properties of Cooked Japonica Rice” by Dai et al. investigated the impact of adding functional components on the quality of traditional products. The authors aimed to determine whether fructooligosaccharide and inulin, two important soluble dietary fibers with prebiotic functions, could interact with rice components and form a hydrogel-like network structure to regulate the texture, processing characteristics, and structural properties of cooked japonica rice. By adding different concentrations (0–10%) of fructooligosaccharide and inulin into two japonica rice varieties, this study systematically evaluated their effects on cooking properties, texture characteristics, pasting behavior, thermal properties, thermo-mechanical properties, molecular structure, and microstructure of cooked japonica rice. They found that both dietary fibers reduced cooking time and decreased hardness, adhesiveness, springiness, gumminess, and chewiness, while improving cohesiveness, resilience, sensory characteristics, and the overall taste of japonica rice gels. Microstructural observations revealed that dietary fiber addition induced thicker matrix walls and enlarged pore structures, resulting in a softer and more uniformly swollen rice matrix. This work introduced prebiotic polysaccharide-based hydrogel regulation as a strategy for designing functional cereal gel systems. Unlike traditional approaches that mainly focus on modifying starch composition or processing conditions, this work demonstrates that soluble dietary fibers can participate in the formation and regulation of internal composite networks within cooked rice matrices.

In “The Influence of Polysaccharides on the Textural Properties and Water Retention Capacity of Animal–Plant Dual-Protein Gels”, Gao et al. investigated an innovative application of polysaccharide-regulated composite gel systems in the development of nutrient-rich texture-modified foods for individuals with dysphagia. To address the challenge of traditional dysphagia foods often suffering from insufficient nutritional density, poor sensory quality, and limited texture adaptability, Gao et al. studied the effects of xanthan gum, konjac glucomannan, and guar gum (0.5%, 1.0%, and 1.5%) on the gel properties, protein conformation, and microstructure of pork–whole soy milk composite gel. Among the tested formulations, the pork–whole soy milk composite gel containing 1.0% xanthan gum exhibited the optimal performance, showing a 43.2% increase in water-holding capacity while maintaining only 23.5% of the hardness of the control gel. The optimized gel met the requirements of both International Dysphagia Diet Standardisation Initiative (IDDSI) Level 5 and Japanese Dysphagia Diet Level III, demonstrating its suitability for dysphagia-oriented food applications. This composite gel simultaneously improves nutritional value, water retention, and swallowing safety after polysaccharide addition. This publication broadens the application of composite gel systems in the food industry by extending their use from traditional applications such as meat products, dairy gels, and delivery systems toward medical nutrition foods and personalized texture-modified foods.

The study “Assessment of Frozen Stored Silver Carp Surimi Gel Quality Using Synthetic Data-Driven Machine Learning (SDDML) Model” by Yang et al. developed an SDDML to overcome the limited experimental dataset commonly encountered in food gel research and to predict optimal additive formulations for improving gel quality. Silver Carp (Hypophthalmichthys molitrix) has considerable potential as a protein resource and exhibits deteriorated gel-forming ability after long-term frozen storage due to protein denaturation and aggregation. To address this limitation, the study explores the effects of functional additives, including manufactured microfiber, transglutaminase, and chicken skin collagen, on restoring the structural stability and textural properties of frozen-stored silver carp surimi gels. It also evaluates the influence of frozen storage duration (0–6 months) and additive incorporation on the quality characteristics of silver carp surimi gels. The results indicate that transglutaminase showed the strongest protective effect, with only 0.1 wt% transglutaminase effectively counteracting the negative influence of prolonged freezing and restoring key textural properties, including gel strength, hardness, and chewiness, to levels comparable with fresh fish samples. Furthermore, the authors established an SDDML model based on different machine learning algorithms to predict gel quality from additive composition. The model closely matched experimental characteristics (R^2^ = 0.871–0.889) and expanded the original experimental dataset from 60 to 240 data points. This publication broadens the application of composite gel systems in the food industry by extending their role from traditional texture modification toward intelligent design, resource valorization, and precision food manufacturing.

The paper “The Thermodynamic and Gelation Properties of Ovalbumin and Lysozyme” by Wang et al. investigates how different processing conditions affect the properties of composite gels. Since egg white proteins are widely used as functional ingredients due to their excellent emulsification, foaming, and gelation properties, controlling their thermal behavior is critical for improving the quality and stability of egg-based foods. The authors aim to provide a deeper understanding of how processing conditions, including pH, protein concentration, heating rate, sugars, and salts, regulate protein unfolding, denaturation, aggregation, and gel formation, thereby improving the design and application of egg-white-protein-based composite gel systems in food processing. They found that different processing conditions regulate the formation of complex gels by altering the conformation of protein molecules. This work establishes a molecular-level regulation strategy for designing composite gel systems through precise control of thermal and environmental factors.

A review, “Food-Grade Emulsion Gels as Nutrient Delivery Systems—Standardized Workflow for Fabrication, Characterization, and Application” by Li et al., was also published in this Special Issue. They investigated the development of a standardized research framework for designing and evaluating food-grade emulsion gels as advanced nutrient delivery systems. The paper systematically summarizes the fundamental principles, preparation methods, and application strategies of food-grade emulsion gels. The authors first discuss the structural characteristics of emulsion gels, which typically consist of emulsifier-coated oil droplets immobilized within a three-dimensional biopolymer network. This unique architecture allows emulsion gels to provide the advantages of emulsions (such as encapsulation of hydrophobic bioactive compounds) and hydrogels (such as structural stability and controlled release properties) simultaneously. The review then presents a standardized workflow for emulsion gel fabrication, emphasizing the importance of formulation parameters including oil phase composition, emulsifier type, biopolymer selection, gelation mechanisms, and processing conditions. Furthermore, the authors propose a multiscale characterization framework involving structural analysis, rheological properties, mechanical properties, thermal stability, water/oil holding capacity, and stability evaluation under food processing and storage conditions. Special attention is given to gastrointestinal digestion models and biological evaluation methods, including cell-based assays, animal studies, and human trials, to assess nutrient release behavior and bioavailability. The standardized workflow proposed in this study provides researchers and food manufacturers with practical guidelines for designing next-generation functional foods containing vitamins, polyphenols, lipids, probiotics, and other bioactive compounds. Furthermore, the framework facilitates the transition of emulsion gel technologies from laboratory-scale studies toward industrial applications by improving consistency, quality control, and regulatory acceptance.

## 3. Conclusions and Outlook

The articles and reviews in this Special Issue offer insightful perspectives and inspiration for the application of composite gels in the food industry. These articles provide the following insights and references for the development of this field.

First, composite gels are multi-component systems, and many factors influence their properties. The source and composition of raw materials, processing conditions, etc., all affect the properties of the components and thus alter the properties of the final product. Therefore, accurately selecting and controlling the properties of different raw materials, and then processing them to produce various composite gels that satisfy consumers, is a current research focus.

Second, with increasing demand from consumers for novel and functional products, adding various non-traditional ingredients to composite gel systems is currently at the forefront of research. How these novel ingredients affect the quality of traditional systems is a key focus. Furthermore, with the acceleration of processing environments and industrialization, the question of how various novel processing methods affect the properties of composite gels is also a current key area of concern for composite gels.

Finally, the functional properties of composite gels are affected by many factors, and how to non-destructively and rapidly evaluate the properties of composite gels using non-traditional methods is also a current research direction. With the opportunities provided by scientific technologies such as AI and scanning electron microscopy, developing precise and rapid evaluation methods is a key focus in the field of composite gels.

In conclusion, the study of the properties of composite gels in the food industry is a cutting-edge research direction. Future research should focus more on developing new resources and applying advanced technologies to create more satisfactory products. Furthermore, with changing market demands and the need for interdisciplinary integration, interdisciplinary collaboration is crucial for the development of composite gel systems in the food industry.
